# Primary pleural leiomyosarcoma with rapid progression and fatal outcome: a case report

**DOI:** 10.1186/1752-1947-6-101

**Published:** 2012-04-05

**Authors:** Ghizlane Rais, Soundouss Raissouni, Houda Mouzount, Meryem Aitelhaj, Siham Khoyaali, Fadoi El Omrani, Hind Mrabti, Ahmed Jelthi, Hassan Errihani

**Affiliations:** 1 Medical Oncology Department, National Institute of Oncology, Rabat, Morocco; 2 Pathology Department, Hôpital Universitaire International Cheikh Zaid, Rabat, Morocco

## Abstract

**Introduction:**

Leiomyosarcomas are neoplasms of smooth muscles that most commonly arise from the uterus, gastrointestinal tract, or soft tissue. Primary pleural leiomyosarcoma is extremely rare. To the best of our knowledge, only nine cases have been published to date. Because of the rarity of pleural leiomyosarcoma and its similarity (clinical and histological) to other pleural neoplasms, particularly sarcomatous mesothelioma, diagnosis is often difficult.

**Case presentation:**

A 58-year-old North African man was admitted with complaints of dyspnea and chest pain to our hospital. Chest computed tomography revealed right pleural effusion and pleural thickening. A transthoracic needle biopsy yielded a diagnosis of leiomyosarcoma, and tumor cells were strongly and uniformly positive for vimentin, a smooth muscle actin at immunohistochemical analysis. A general examination did not show any metastatic lesions in other areas. One month after diagnosis, the tumor grew rapidly, with pulmonary invasion, and therefore he was treated only by palliative care. He died from respiratory failure one month later. Because no organ of origin of the leiomyosarcoma, other than the pleura, was detected, this case was diagnosed as a primary pleural leiomyosarcoma.

**Conclusions:**

Although leiomyosarcoma originating from the pleura is rare, this entity is increasingly described. The purpose of presenting this case report is to raise awareness among clinicians to consider this clinical entity as a differential diagnosis when a pleural mass is identified.

## Introduction

Leiomyosarcomas are cancers of smooth muscle cells that can arise from any location but occur most often in the uterus, retroperitoneum, or intra-abdominal region. Primary pleural leiomyosarcomas are extremely rare [1,2]. There are few reports of individual cases [1-5], but no large series from a single institution has been published. On radiological examination, primary pleural leiomyosarcoma presents as a mass or a pleural effusion. Metastasis is uncommon and typically occurs late in the disease process, underscoring the importance of early detection. When primary pleural leiomyosarcoma is seen in the pleura, it is important to establish whether the tumor originated in the lung or is secondary to metastases from a separate location, the latter of which is much more common.

To definitively establish the pleura as the primary site for leiomyosarcoma, all other possible sites of origin within the body must be excluded. Therefore, diagnosis is difficult and management is complete resection of the tumor if feasible [4,6,7]. In this report, a rare primary pleural leiomyosarcoma exhibiting rapid growth and fatal outcome is described. The diagnostic approach to our patient is presented and the pertinent literature is reviewed.

## Case presentation

**Figure 1:**
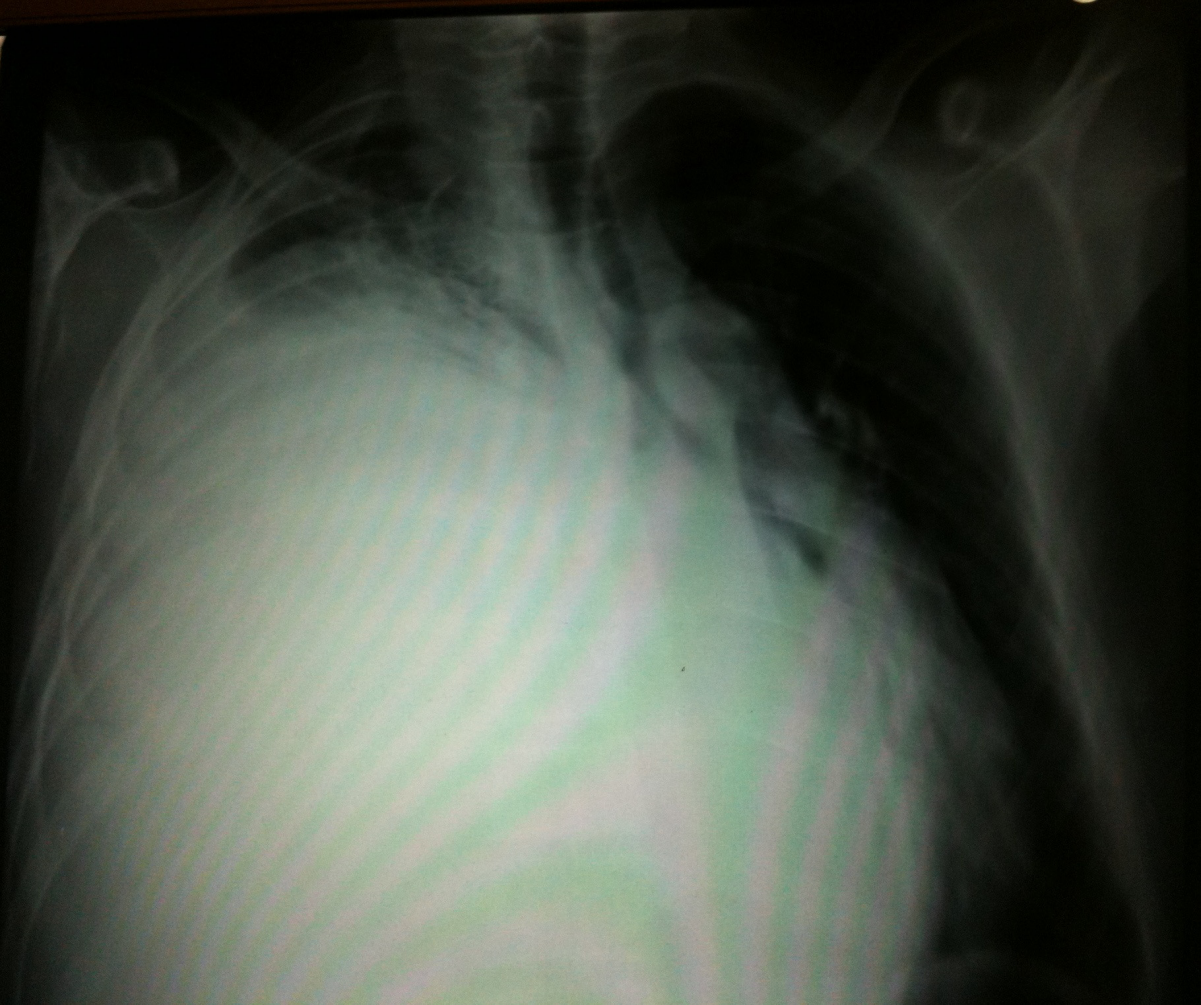
**Chest radiograph (postero-anterior view) shows the presence of a right-sided opaque hemithorax**.

**Figure 2:**
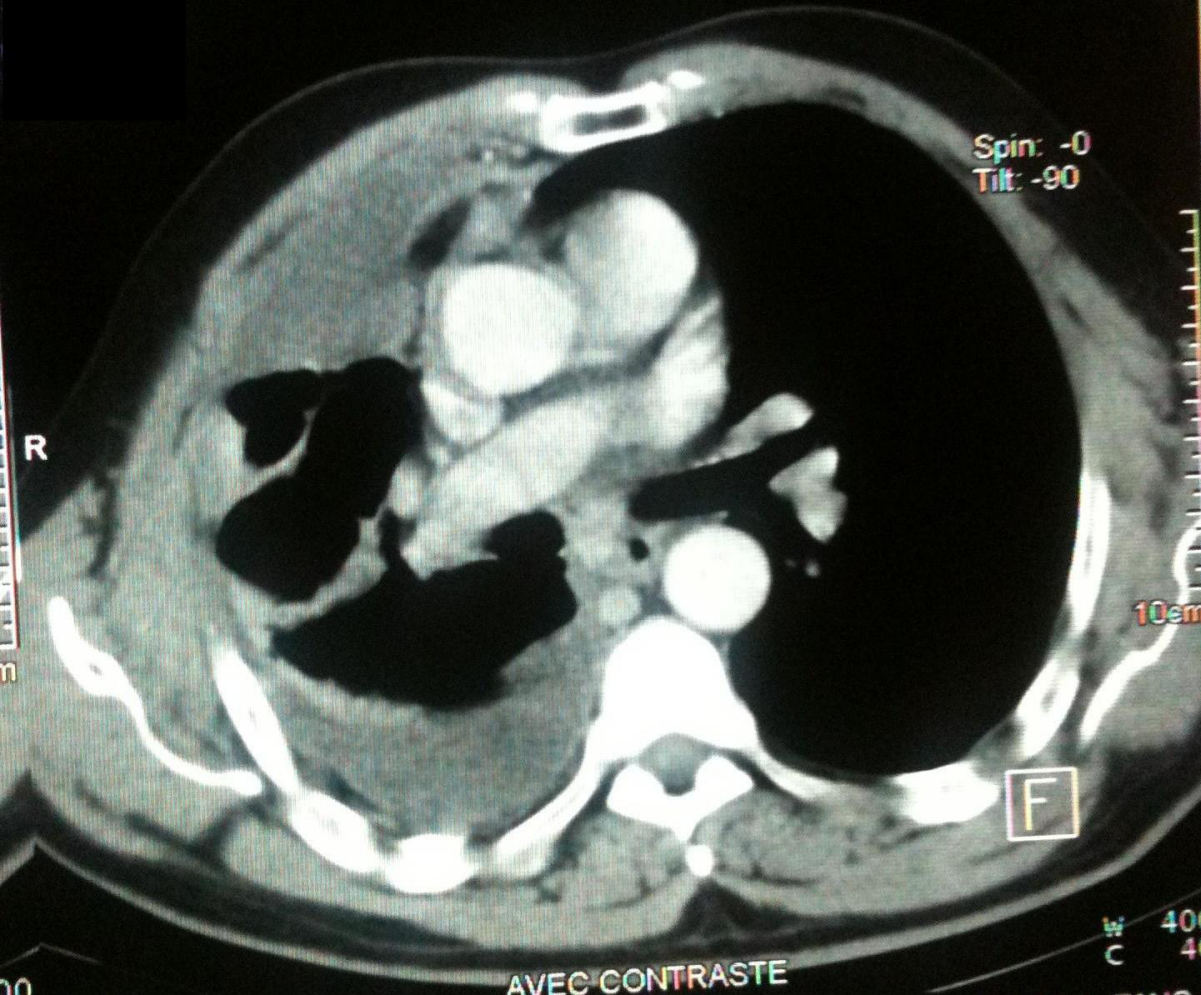
**Selected sections of a conventional computed tomography scan of the chest**. The scan shows a right-sided cavity with thick and irregular walls in the right lower lobe, pleural effusion, and a partially collapsed right lung.

**Figure 3:**
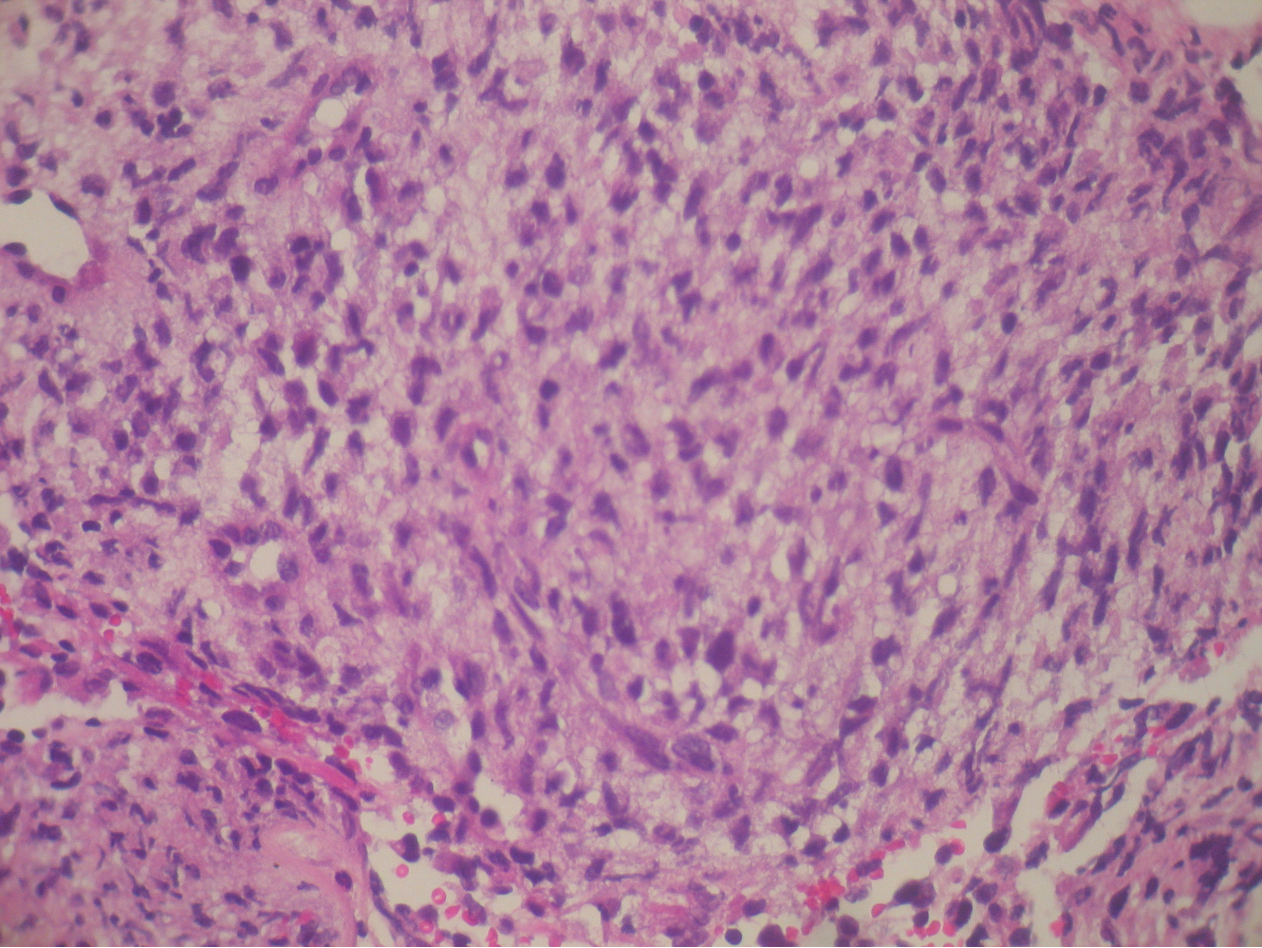
**Biopsy shows neoplastic spindle cell proliferation arranged in interlacing bundles and fascicles**. Stain: hematoxylin and eosin; magnification: 40×.

**Figure 4:**
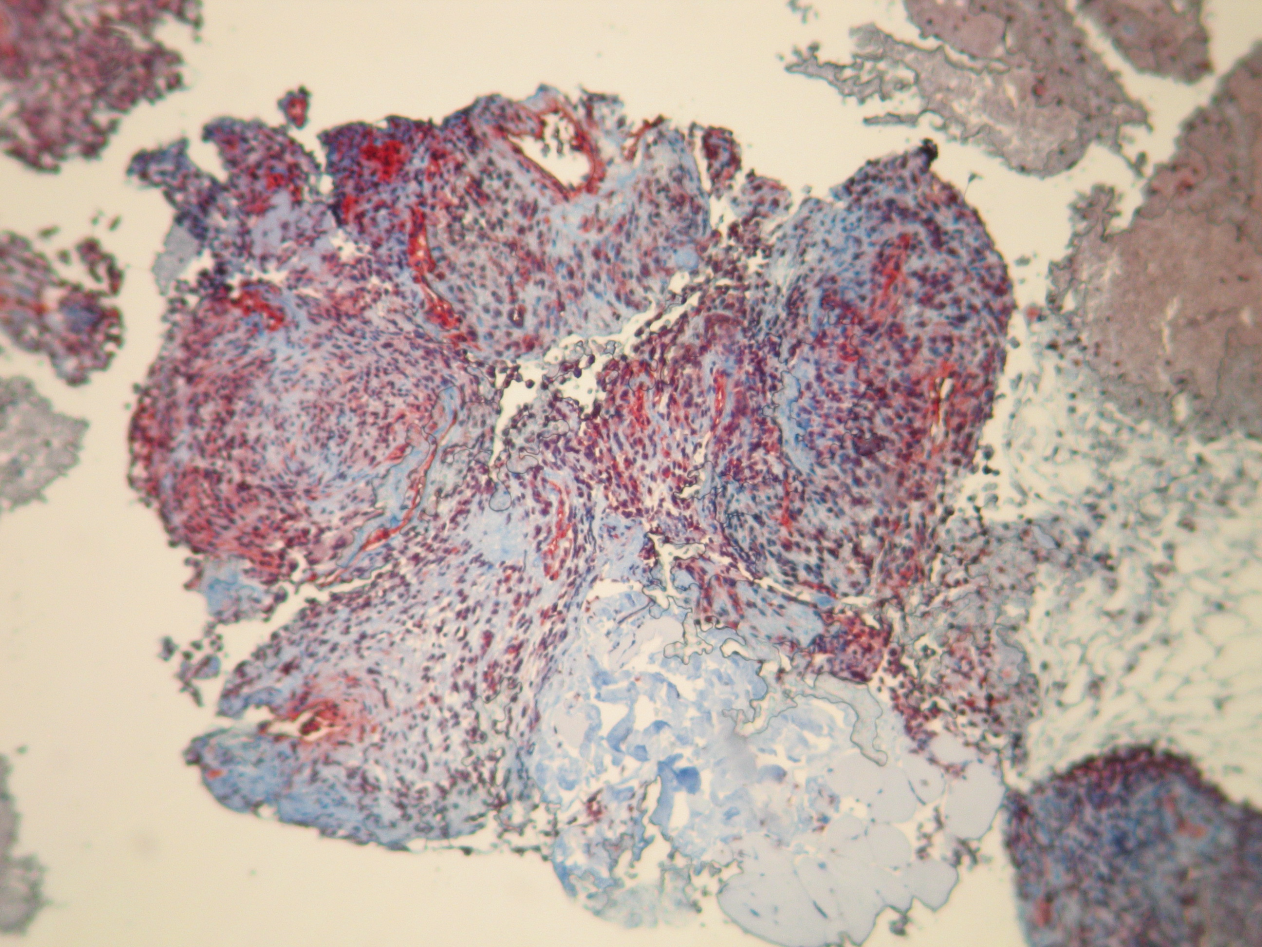
**Tumor cells show strong positivity for anti-acute myeloid leukemia**. Stain: immunohistochemical; magnification: 20×.

**Figure 5:**
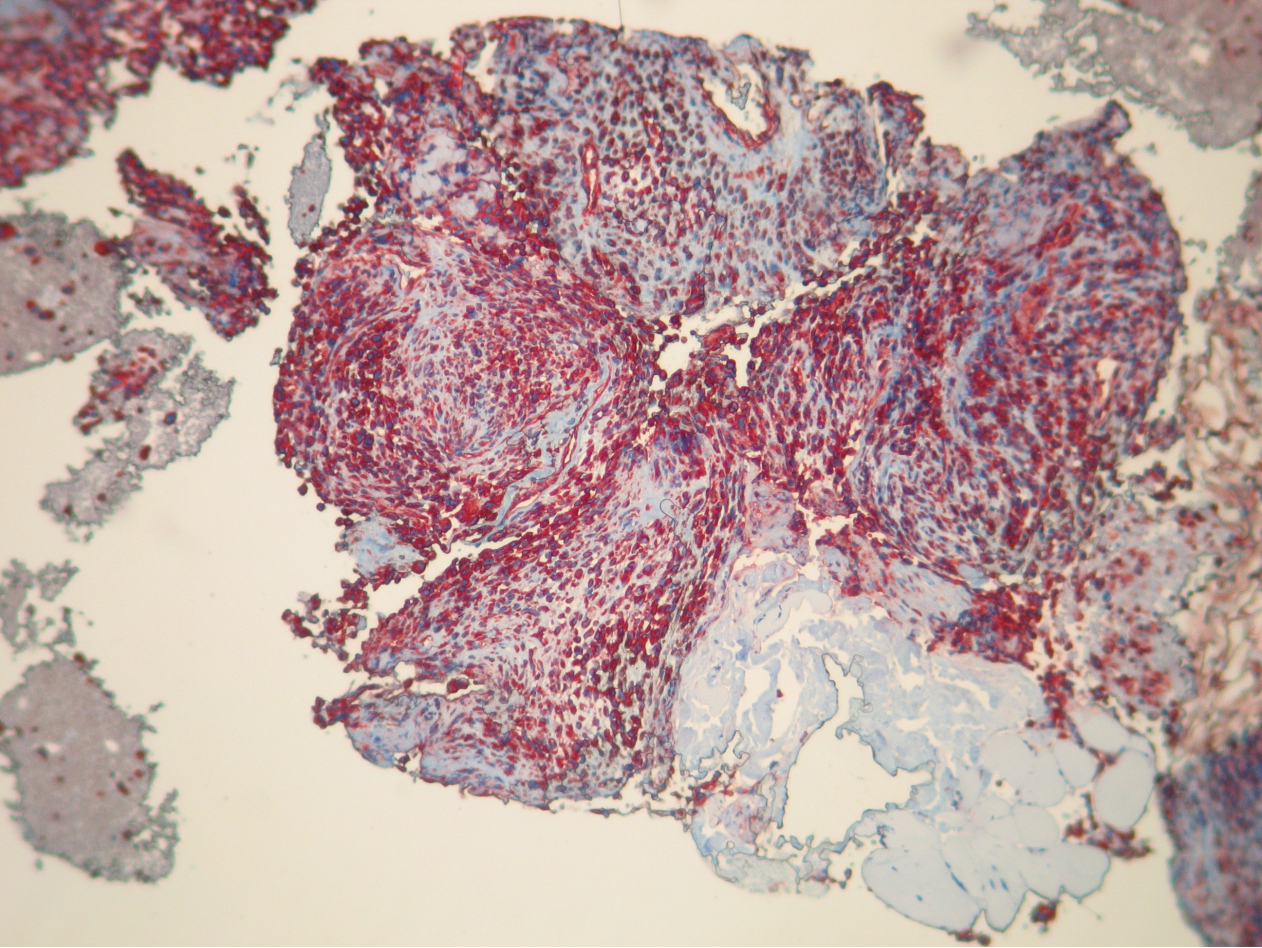
**Immunohistochemical staining reveals diffuse and strongly positive reactions with anti-vimentin antibodies**. Magnification: 20×.

**Figures 6:**
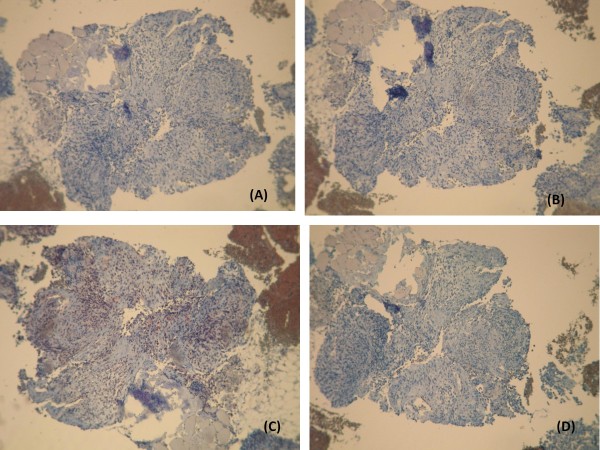
**Immunohistochemistry shows that the tumor cells are negative for cytokeratin 7 (A), cytokeratin 20 (B), calretinin (C), and PS-100 (D)**. Magnifications: 200×.

**Figure 7:**
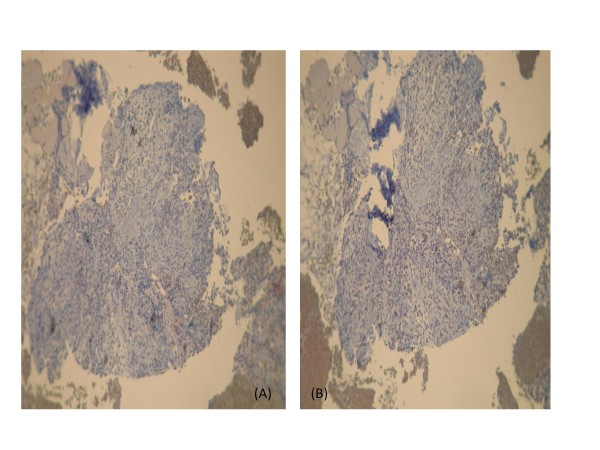
**Immunohistochemistry shows that the tumor cells are negative for CD-117 (A) and CD-34 (B)**. Magnifications: 200×.

A 58-year-old North African man presented with complaints of dyspnea and right-sided chest pain for the previous two months. He had a 25-year history of tobacco use, smoking one pack of cigarettes per day for 25 years, but had no additional risk factors. Three liters of blood-stained fluid was removed from the right side of his chest in another hospital and then was referred to our hospital for further evaluation and management. A biochemical analysis of the pleural fluid revealed that the nucleated cell count was 7800/mm^3 ^(23% neutrophils and 71% lymphocytes), total protein concentration was 32 g/dL (a pleural fluid-to-serum ratio of 0.52), lactate dehydrogenase level was 460 IU/L, and glucose level was 110 mg/dL. The pH of the pleural fluid was 7.30. These findings were consistent with exudative pleural effusion. The results of pleural fluid cytology were negative for malignant cells. The pleural fluid was cultured for bacteria (aerobic and anaerobic), fungi, and mycobacteria, and the results were negative. During a clinical examination, the breath sounds were decreased on the right side. A chest radiograph showed the presence of an opaque right hemithorax, and the mediastinum was pushed toward the right (Figure 1). Computed tomography (CT) of the chest revealed a right-sided cavity with thick and irregular walls extending throughout the pleural space in the right lower lobe, pleural effusion, and a partially collapsed right lung (Figure 2). An interventional radiologist performed a transthoracic needle pleural biopsy, which revealed a mesenchymal lesion consistent with a smooth muscle cell-type lesion. On microscopic examination, the tumor consisted of neoplastic spindle cell proliferation arranged in interlacing bundles with hyperchromatic nuclei and frequent mitoses (greater than 10 mitoses per 10 high-power fields) (Figure 3). No areas of hemorrhage or necrosis were seen. Immunohistochemistry (IHC) staining was performed. The panel of monoclonal antibodies consisted of cytokeratin 5/6, 7, and 20, vimentin, smooth muscle actin, calretinin, CD-117, TTF1, EMA, CD-34, and S-100 protein. The tumor cells were positive for vimentin and smooth muscle actin strongly and uniformly (Figures 4 and 5). These cells were immunonegative for all other markers tested, ruling out carcinoma (cytokeratin), sarcomatous mesothelioma (calretinin), solitary fibrous tumor (CD-34), and neurogenic sarcoma (S-100 protein) (Figures 6 and 7). After the pathological diagnosis, a CT scan of the whole body was performed but did not reveal any tumors. The authors diagnosed the lesion as primary pleural leiomyosarcoma. However, the tumor grew rapidly, with pleural effusion. Our patient was treated only by palliative care. He died one month later due to acute respiratory failure.

## Discussion

Leiomyosarcomas are neoplasms of smooth muscles that most commonly arise from the uterus, gastrointestinal tract, or soft tissue. Pleural leiomyosarcomas originating in the serosal membranes are even more unusual and represent controversial entities [1-3]. Sometimes, these tumors are classified as sarcomatous types of mesothelioma when they are diffuse and as variants of solitary fibrous tumors when they are localized [3,5].

To the best of our knowledge, no large series has documented the exact number of pleural leiomyosarcoma cases worldwide. Data are available in isolated case reports and small case series. To the best of our knowledge, fewer than 10 cases have been reported [4-6]. Thanks to increasing awareness of this entity and increasing application of adjunctive diagnostic tools, more cases will be diagnosed in the future.

Clinically, pleural leiomyosarcoma resembles other primary tumors of the pleura in symptomatology (dyspnea, chest pain, or cough), physical signs, and radiology [5,6]. On radiological examination, a mass lesion or pleural effusion or both are common, as in our case. In some cases, the tumor encases the lung, similarly to mesothelioma [5,7].

Extrathoracic primary tumors should be excluded by physical examination and CT. Most pleural leiomyosarcomas are metastatic from other sites, including the gastrointestinal tract, uterus, retroperitoneum, and lung [1-4]. So exclusion of metastatic leiomyosarcoma in the pleura is an essential step in diagnosing the primary lesion. In all reported cases, a localized unilateral pleural lesion with no abnormality detected on careful examination of the soft tissue, gastrointestinal tract, and genitourinary tract suggested primary disease of the pleura [2-7]. The lesion in this case report is primary pleural leiomyosarcoma because imaging modalities, including CT, revealed no tumors other than the pleural tumor.

On reviewing the literature on pleural leiomyosarcoma, we found that most of the cases were diagnosed by surgical biopsy at either thoracoscopy or thoracotomy [1,3]. Occasionally, the diagnosis has been made by CT-guided transthoracic biopsy, according to isolated reports [4,7]. Histological examination supplemented with IHC staining studies is the most reliable and conclusive method of diagnosing leiomyosarcoma and differentiating it from other more frequent primary pleural malignancies, such as malignant mesothelioma (sarcomatous), malignant fibrous histiocytoma, solitary fibrous tumor of pleura, synovial sarcoma, and neurogenic tumors.

Microscopically, leiomyosarcomas are characterized by malignant spindle cells with cigar-shaped nuclei and scant fibrillary cytoplasm. Variable mitotic activity is observed [1,4,5]. However, because of the rarity of pleural leiomyosarcoma and its similarity (clinical and histological) to other pleural neoplasms, particularly sarcomatous mesothelioma, pathological diagnosis is often difficult.

Therefore, the use of an antibody panel is recommended in order to confirm the diagnosis of pleural leiomyosarcoma and to rule out a commonly considered differential diagnosis. Immunohistochemically, leiomyosarcomas are nearly uniformly positive for smooth muscle actin, desmin, and vimentin and negative for calretinin, carcinoembryonic antigen, cytokeratin, leukocyte common antigen, neuroendocrine filament, CD-117, and S-100 protein [4,5]. In our case report, histological and immunohistochemical characteristics of the tumors studied were basically similar and conformed to those described by other authors.

Histopathology and IHC staining have been supplemented by cytogenetic analyses, which can confirm the diagnosis of leiomyosarcoma [5]. Cytogenetic studies of the smooth muscle tumor of the pleura have revealed contained chromosomal losses at 10q and 13q, which are the most common losses described in soft tissue leiomyosarcomas [8].

The optimal treatment for pleural leiomyosarcoma has not been defined. Surgery is the mainstay of therapy for pleural sarcoma in limited disease, and even in some cases having local spread, resection was feasible [1,6,7,9]. Multi-modal therapy, including surgery and radiation, has also been used. The need for adjuvant radiation or chemotherapy may be assessed on a case-by-case basis depending on the histological grade of the tumor and the clinical stage at the time of diagnosis [9]. In the literature, the most common treatment is surgical resection alone or surgery with adjuvant radiation therapy [4-7].

As with leiomyosarcomas of other soft tissue sites, histological grade and clinical stage of the disease are the best prognostic factors. Patients with unresectable sarcoma or metastatic disease are treated with chemotherapy [10]. Leiomyosarcomas are chemosensitive to doxorubicin, ifosfamide, and trabectedin, and the overall response rate is approximately 20% [11]. The median survival for patients with extensive disease is about 12 months [10,11]. In our case report, pleural leiomyosarcoma was misdiagnosed as pleural tuberculosis because of the frequency of this infection in Morocco. The diagnosis of pleural leiomyosarcoma was made later, after deterioration of the general condition of our patient and aggressive progression of the tumor, which was unresectable.

## Conclusions

The addition of our case to the literature offers new clinicopathological data useful for better defining the diagnosis and biological behavior of pleural leiomyosarcoma. The purpose of presenting this case report is not only to report an uncommon pleural tumor but also to raise awareness among clinicians adding this clinical entity as a differential diagnosis when a pleural mass is identified.

## Abbreviations

CT: computed tomography; IHC: immunohistochemistry.

## Consent

Written informed consent was obtained from the patient's next of kin for publication of this case report and any accompanying images. A copy of the written consent is available for review by the Editor-in-Chief of this journal.

## Competing interests

The authors declare that they have no competing interests.

## Authors' contributions

GR was involved in the analysis of the data and the literature search and wrote the manuscript. SR helped with the patient management and revision of the manuscript. H Mouzount and MA helped with the literature research. H Mrabti, SK, and FE helped with modifications and revision of the manuscript. AJ performed the histological examination. HE approved the treatment and analyzed the literature data. All authors read and approved the final manuscript.
